# Clinical predictors of poor outcome of bacterial meningitis in infants less than 90 days: a systematic review

**DOI:** 10.3389/fped.2024.1414778

**Published:** 2024-09-19

**Authors:** Ying Liu, Yu Feng, YanPing Guo, JingJing Chen, Chang Liu, JiaBi Liang

**Affiliations:** Department of Pediatrics, Peking University Shenzhen Hospital, Shenzhen, China

**Keywords:** infants, bacterial meningitis, prognostic factors, risk factors, systematic review

## Abstract

**Background:**

bacterial meningitis (BM) is more common in infants than at any other time in life and remains a devastating disease with considerable risk of death and morbidity. This article aims to gather the currently available evidence to perform a systematic review of clinical factors that may predict or be associated with BM death and/or sequelae in infants < 90 days of age.

**Methods:**

The Medline/PubMed, Cochrane Library and Embase databases were systematically searched for prognostic studies that described risk factors for mortality and sequelae in infants aged <90d with BM. The databases were searched from the beginning of the database to December 31st, 2022.The quality of cohort studies was assessed by the Newcastle-Ottawa Scale (NOS). The quality of cross-section studies was assessed by the Agency for Healthcare Research and Quality (AHRQ). A systematic review was undertaken to ascertain the prognostic factors proven to be noteworthy.

**Results:**

Of the 1,431 studies retrieved, 20 were eligible for the final analysis including 11 cohort and 9 cross-sectional studies were identified. Four risk factors predicting poor outcome were mentioned mostly in those studies, including prematurity or low birth weight (LBW), seizures, coma, and elevated CSF protein. But only preterm, coma and elevated CSF protein were identified by multivariate analyses in more than one study.

**Conclusions:**

This study demonstrates several potential predictive factors to the poor outcomes of BM in infant. But with large heterogeneity, these predictors should be evaluated by further well-designed prospective studies.

**Systematic Review Registration:**

https://www.crd.york.ac.uk/, identifier CRD42017074949.

## 1 Introduction

Bacterial meningitis (BM) is more common in young infants than at any other time of life and remains a devastating disease with considerable risk of mortality and morbidity (1). The incidence of BM is 20/100,000 in infants under 2 years old, and 400/100,000 in newborns (2). In infants aged <90 days, the annual incidence of BM was 0.38 per 1,000 live birth (3). The mortality of BM in neonates ranged from 6% to 15% in developed and 25% to 58% in low-MICs, respectively, and as many as 20 to 58% of survivors are prone to permanent neurological sequelae (3–7). Early identification of risk factors will be of great significance for clinical decision-making, planning of follow-up schedule, and early intervention (1). Hence, this article was performed to collect the currently available evidence to provide a systematic review of the clinical factors that may predict or relate to death and/or sequelae of BM in infants aged <90 days.

## 2 Material and methods

This systematic review was conducted following the Cochrane Collaboration and Preferred Reporting Items for Systematic Reviews and Meta-Analyses (PRISMA) guidelines (8, 9). The PRISMA checklist is included in Supplementary S1. This study is registered with PROSPERO (CRD42017074949) (9).

### 2.1 Search strategy

The Medline/PubMed, Cochrane Library and Embase databases were systematically searched for prognostic studies that described risk factors for mortality and sequelae in infants aged <90d with BM. The databases were searched from the beginning of the database to December 31st, 2022. The key words used as search terms were “neonate,” “infant”, “bacterial meningitis,” and “prognostic factors” “predictor” and “sequelae” or “outcome”. The search strategy is listed in the Supplementary Data Sheet. The references cited in each of the selected studies were checked to acquire relevant studies that had not been identified by the above-described retrieval methods.

### 2.2 Inclusion and exclusion criteria

To be included, studies had to report original data on describing risk factors for outcome of BM (other than tuberculous meningitis) occurred within 90 days after birth. The results were published in English, and the full-text article could be retrieved. Studies aimed at identifying the risk factors (except for biomarkers in genes) for a poor prognosis including death and/or sequelae (not only short term complications) were defined prior to our study. Cohort studies and cross-section studies in which Odds Ratios or *P* values for the relationship between prognostic factors and outcomes were provided were included. Studies were excluded (1) if they were another article type (i.e., expert opinions, letters to the editor, editorials, comments, narrative reviews, and case reports), or (2) Not running statistical analysis of prognostic factors and outcomes, or (3) Not analyzing the relationship between prognostic factors and outcome, or (4) if they were with a small sample size (<50 subjects).

### 2.3 Data selection

Full-text articles were identified following a preliminary screening of titles and abstracts and were reviewed in detail by two researchers separately, with a third researcher resolving any disagreements if required. For each selected article, two researchers extracted the following data to an excel spreadsheet including study characteristics (country, design, study period, and statistical method), study population (case definition, exclusion criteria, sample size, age, Follow-up years and outcome [(1) mortality (2) sequelae and (3) poor outcomes (when no distinction between mortality and sequelae was drawn in the original study).], and significant prognostic factors. The final scope of the articles and all correlative data or information were discussed at regular meetings. All data included in this article were confirmed by all authors.

### 2.4 Risk of bias assessment

The quality of the cohort studies was assessed using the Newcastle-Ottawa Scale (NOS). We used 3 groups (selection, comparability, and outcomes) and 8 projects to judge their quality. A figure is presented to show the risk of bias in a cohort study performed using the NOS. The quality of the cross section studies was assessed by the Agency for Healthcare Research and Quality (AHRQ) scale, which contains 11 items. An item was scored “0” if it was answered with “NO” or “UNCLEAR” and “1” if it was answered with “YES.” Article quality was assessed as follows: low quality = 0–3, moderate quality = 4–7, and high quality = 8–11.

### 2.5 Statistical analyses

These prognostic factors were manually classified and summarized. The prognostic factors that were identified as significant in over four studies were ultimately incorporated into the systematic review. Review Manager (RevMan) 5.3 software (10) was used for data analysis. Studies included needed to have reported OR and corresponding standard errors, or 95% confidence intervals. The natural log of OR and standard errors, were calculated for each study independently, then pooled and weighted by generic inverse variance to provide an overall OR, 95% confidence interval, and *p* value.

The I^2^ statistic the chi-squared statistical test, and the corresponding *p* value were determined automatically by the programme and displayed in a forest plot. Heterogeneity was low (0∼40%), moderate (30∼60%), substantial (50∼90%), or high (75∼100%). with *p* ≤ 0.01 considered for statistically significant heterogeneity.

## 3 Results

### 3.1 Study characteristics

**Figure 1:**
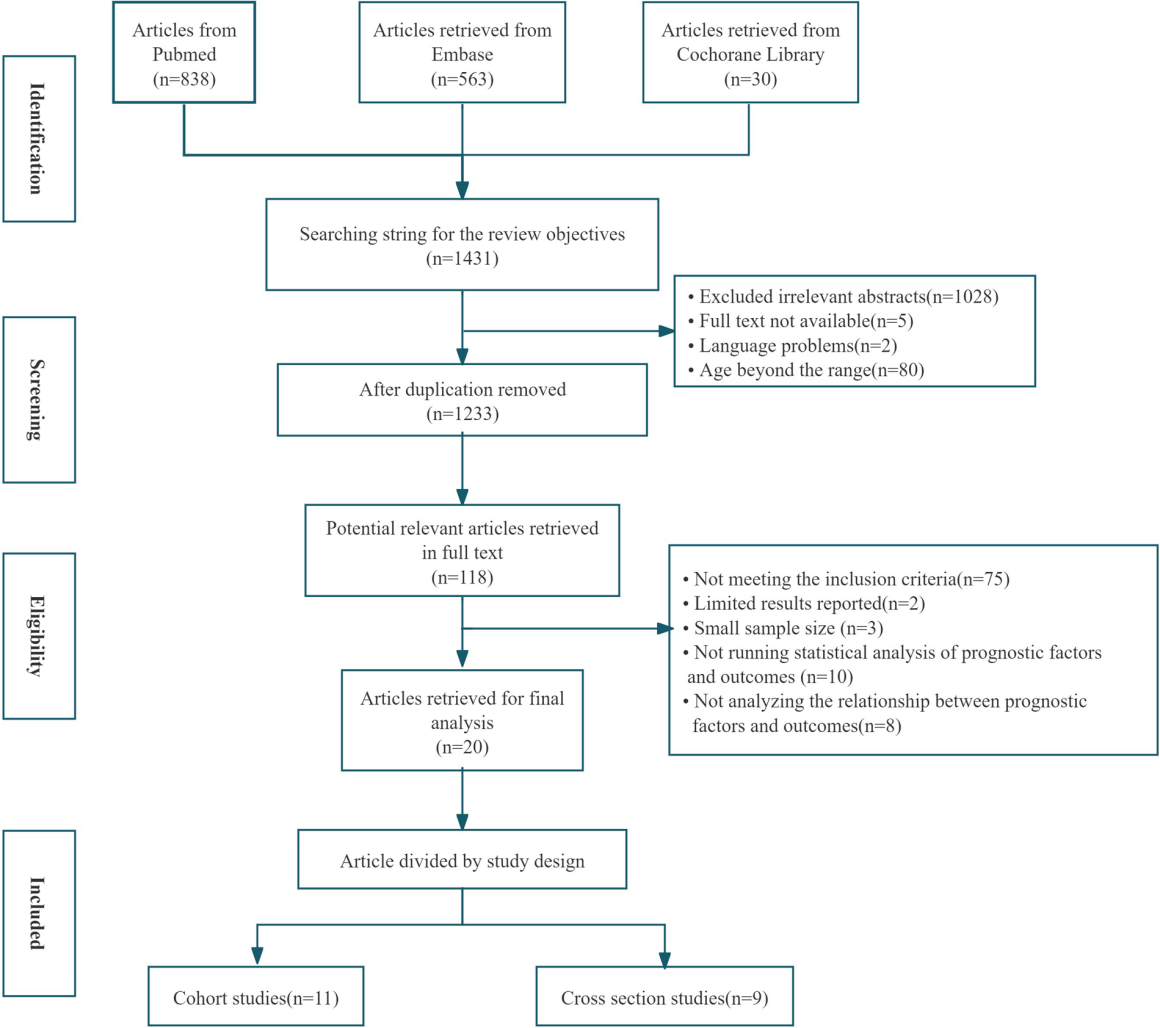
Flow path of the selection.

Out of a total of 1,431 unique records screened, 119 articles seemed to have high potential to meet the inclusion criteria. After we read the full text of and carefully screened each article, 99 of the articles were excluded (Flow Path of the Selection was showed in Figure 1). Finally, 20 articles were identified. Studies were grouped by design into cohort studies (*n* = 11) and cross-sectional studies (*n* = 9). The study characteristics of all if the included publications are summarized in Tables 1, 2. 17 studies with populations aged from 0 to 31 days reported the mortality of 3.0%–48% and sequelae of 19.3%–39%. 3 studies of infants within 0–90 days had the mortality of 7%–9.2% and sequale of 74%.

**Table 1:** Study characteristics and quality assessments for cross studies.

Study	Country/region	Year	Study design	Statistical method	Object	*N*	Outcome	AHRQ
Bortolussi et al. (11)	Australia	1968–1974	Retrospective, single centre	Univariate	0–31d	52	Mortality (48%)	8
Mulder et al. (12)	Netherland	1976–1982	Retrospective, single centre	Univariate	0∼27d	68	Mortality (25%)	6
Nathoo et al. (13)	Zimbabwe	7	Retrospective, single centre	Univariate	0∼28d	94	Mortality (41%)	7
Gebremariam (14)	Ethiopia	1987–1996	Retrospective, single centre	Univariate	0∼28d	55	Mortality (40%)	7
May et al. (15)	Australia and New Zealand	1992–2002	Prospective, multicentre	Multivariate	0∼28d	78	Mortality (6.5–33.6%)	8
Krebs et al. (16)	Brazil	1994–2004	Retrospective, single centre	Univariate	0∼28d	87	Mortality (11.5%)	7
holt et al. (17)	English	1996–1997	Prospective, multicentre	Multivariate	0∼28d	274	Mortality (6.6%)	9
Gaschignard et al. (7)	French	2001–2007	Prospective, multicentre	Multivariate	0∼28d	444	Mortality (13%)	9
Basmaci et al. (18)	France	2001–2013	Prospective, multicentre	Univariate	14（0∼89)d	325	Mortality (9.2%)	8

**Table 2:** Study characteristics of cohort studies.

Study	Country/region	Year	Study design	Statistical method	Object	*N*	Outcome
Klinger et al. (19)	Canada	1979–1998	Retrospective, single centre	Univariate	0∼28d, GA ≥ 35 w	101	Mortality 12.9%, Sequelae 19.3%
Lin et al. (20)	China, aiwan	1984–2008	Retrospective, single centre	Multivariate	0∼28d, term	156	Mortality 14.8%, sequelae 24.2%
Chang et al. (21)	China, Taiwan	1986–2001	Retrospective cohort study	Univariate	0∼28d	60	Mortality 6.7%, severely abnormal 41.1%
Kamoun et al. (22)	Tunisia	1990–2012	Retrospective, single centre	Univariate	0∼28d	55	Mortality 40%, Sequela 27.3%
Daoud et al. (23)	Jordan	1992–1994	prospective, double centre	Multivariate	0∼28d	53	Mortality 32%, Sequela 39%
Krebs et al. (16)	Brazil	1994–2004	Retrospective cohort study	Univariate	0∼28d	87	Mortality: 11.5%.
Haffner et al. (24)	American	2005–2017	Retrospective cohort study	Univariate	0–30 d, GA ≥ 35 w	103	Mortality: 8.7%, Sequela 30%
Tan et al. (25)	China	2008–2014	Retrospective, multicentre	Multivariate	0∼28d, term	232	Mortality 3.0%, poor outcome: 28.0%^a^
Okike et al. (26)	British	2010–2011	Prospective, multicentre	Multivariate	0∼90d	263	Mortality (9%)
Ouchenir et al. (27)	Canada	2013–2014	retrospective, multicentre	Univariate	0–90d	113	Mortality: 7%, sequale: 74%
Kumar et al. (28)	India	NA	prospective multicentre	Univariate	0∼28d, term	89	Mortality: 11.2%, sequale: 22%

GA, gestational age.

^a^ Including death.

### 3.2 Risk of bias

**Figure 2:**
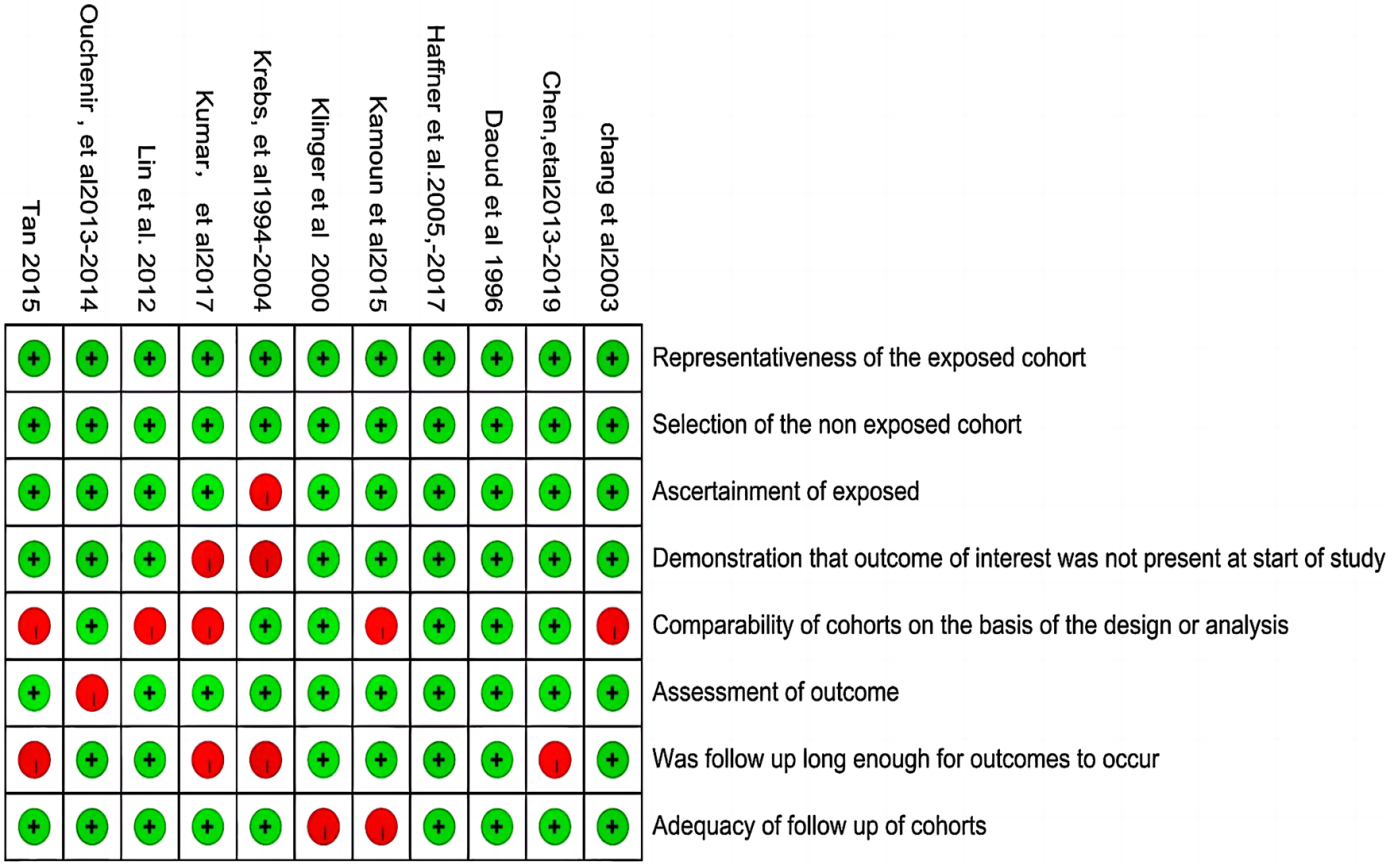
Quality assessments of cohort studies.

The risk of bias of the studies is shown in Table 1 and Figure 2. All of the included studies were moderate/high quality. But there were still some factors affecting the quality assessment. Only a few studies adopted the same diagnostic criteria of BM based on positive culture CSF, and the large heterogeneity diagnostic thresholds for BM and definitions of poor outcomes (listed in Supplementary S2) suggested that there was potential for selection and comparability bias. Four studies acquired the results by means of questionnaires sent to the paediatrician, suggesting the potential for recall bias. Moreover, only 2 of the 11 cohort studies were prospective studies, and more than half of the included studies insufficiently controlled confounding factors, further suggesting the potential for confounder bias.

### 3.3 Pathogens

**Figure 3:**
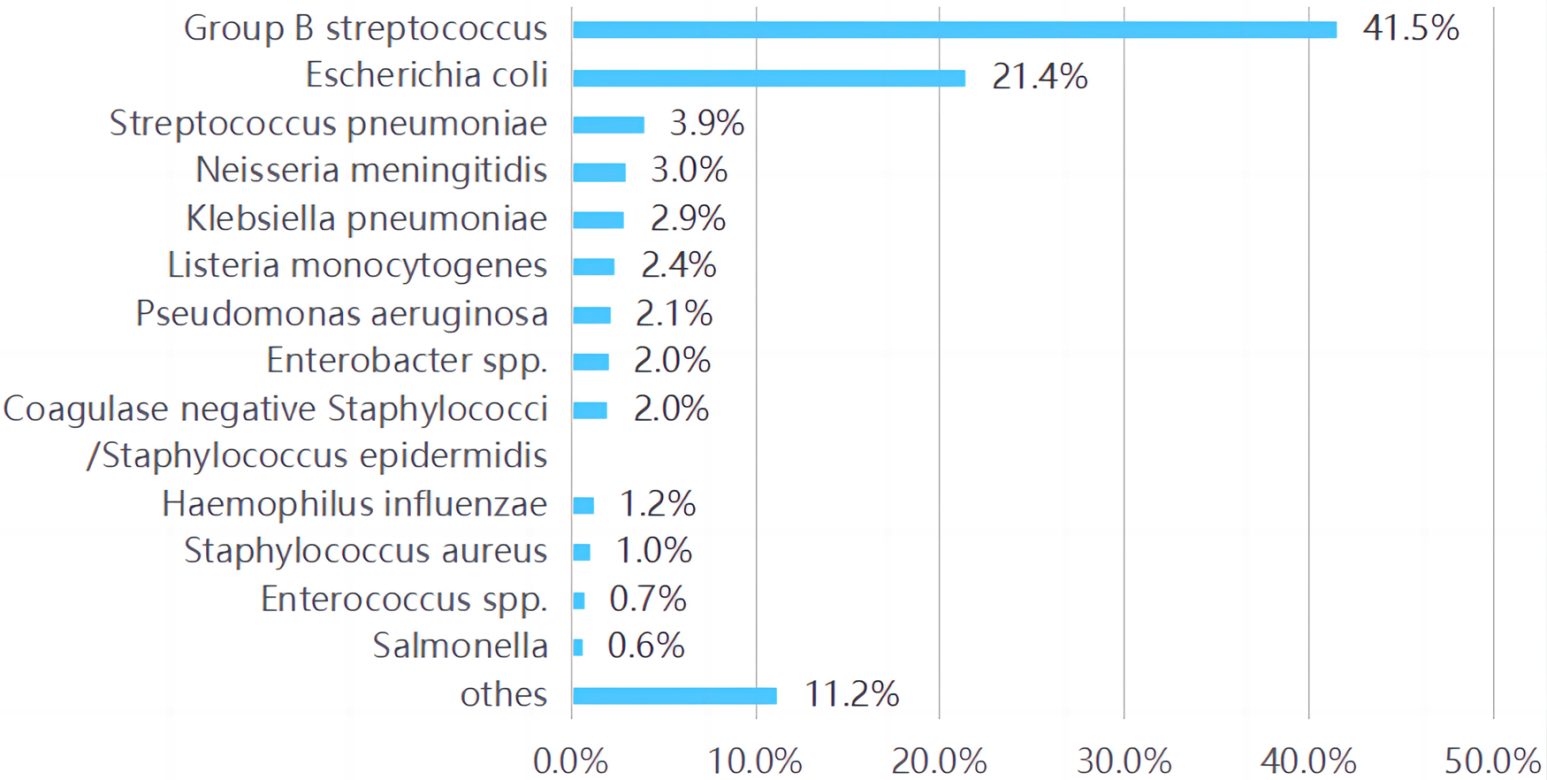
Bacterial pathogens distribution summarized in all the studies involved.

Various pathogen species were described in 17 included articles (of the rest three articles, one only for Group B Streptococcus (GBS) (17), one for coli (18), and one without pathogen analysis). The top three pathogens isolated in all these studies are listed in Supplementary S2. GBS (25%∼62%) and Escherichia coli (E. coli, 10∼46%) were reported the two main pathogens in most studies. When the pathogens in all these studies were summarized, the main were also found to be GBS (41.7%, 858/1,984) and E. coli (21.4%, 443/1,984) followed by Streptococcus pneumoniae (S. pneumoniae, 3.9%, 81/1,984) and Neisseria meningitidis (N. meningitidis, 3.0%, 61/1,984). As shown in Figure 3.

### 3.4 Prognostic factors for poor outcome

Of the cross studies, the major poor outcomes were mortality. Of the cohort studies, the follow up time varied from 1.0 months to 11 years, and the major follow-up outcomes were sequelae or/and mortality. While, there were differences among the studies in diagnostic criteria, definitions of poor outcome, as described in Supplementary S2.

A total of 26 factors were found to potentially predict prognoses, and 14 prognostic predictors were identified by multivariate analyses. We summarized and categorized those factors in Table 3.

**Table 3:** Predictors of poor outcome of infants with BM by multivariate analyses.

Predictors	Studies	Poor outcome	OR (95% CI), *P* < 0.05
Demographic characteristics			
Preterm	Okike et al. (26)	Mortality	5.84 (2.02–16.85)
	Gaschignard et al. (7)	Mortality	2.97 (1.62–5.45)
Birth weight < 1,500 g	May et al. (15)^.^	Mortality	7.2 (4.8–10.9)
Clinical signs			
Poor feeding	Tan et al. (25)	Mortality + sequelae	3.83 (1.22–12.05)
Seizures	Lin et al. (20).	Sequelae	10.10 (2.11–48.32)
Coma	Okike et al. (26)	Mortality	31.85 (8.46–119.81)
	Holt (17)	Mortality	11.14 (3.01–40.87)
Bulging anterior fontanelle	Daoud et al. (23)	Mortality	7.7 (1.7–35.4)
Altered sensorium	Daoud et al. (23)	Sequelae	6.1 (1.4–26.9)
Treatments			
Treated with steroids	Holt (17)	Mortality	5.09 (1.38–17.94)
Laboratory examinations			
Cerebrospinal fluid protein (g/L)	Tan et al. (25)	Mortality + sequelae	4.07 (2.33–7.11) cutoff value: 1.88 g/L
	Lin et al. (20).	Sequelae	171.18 (25.6–1,000) cutoff value: 5.00 g/L
Blood Hemoglobin (g/L)	Tan et al. (25)	Mortality + sequelae	0.62 (0.37–1.04)
Accessory examination			
Hearing impairment	Lin et al. (20).	Sequelae	23.40 (3.62–151.25)
Type of pathogen			
Gram-negative bacterium	May et al. (15)	Mortality	3.3 (2.2–4.9）
Concomitant disease			
Congenital heart disease	Lin et al. (20)	Sequelae	48.96 (6.06–395.64)
Pneumonia	Tan et al. (25)	Mortality + sequelae	3.37 (1.15–9.84)

The others obtained from univariate analyses included: early onset meningitis (<1w) (20), abnormal examination on presentation (24), abnormal neurological examination at discharge (28), pressors/inotrope(s) (19, 24), low cerebrospinal fluid (CSF) glucose (22, 24), positive CSF culture (16), serum WBC (24), neutropenia (18), thrombocytopenia (18, 21), abnormal imaging (24), hydrocephalus on neuroimaging (27), abnormal EEG (28). There were four prognostic factors were found to be significant in more than four studies, including preterm or low birthweight (LBW) (seven studies), seizures (four studies), coma (four studies) and elevated CSF protein (five studies). But only preterm, coma and elevated CSF protein were identified by multivariate analyses in more than one study, as shown in Table 3.

## 4 Discussion

To our knowledge, this is the first systematic review of BM in infants aged <90 days to evaluate the prognostic predictors. Preterm/LBW, convulsions, coma, and elevated CSF protein were most mentioned valuable predictors for poor outcomes. Further analysis also showed preterm/LBW as a good predictor for death. In a systematic review of predictors for poor outcomes in neonatal BM by Mao et al. (29), seizure and high protein levels in CSF were also identified.

### 4.1 Preterm or LBW

In this study, preterm, usually associated with LBW (birth weight < 2,500 g), was significantly correlated with poor outcome, which was involved in seven pervious studies. Furthermore, the risk increased with the degree of prematurity. Okike (26) studied through British Paediatric Surveillance Unit and revealed that premature birth was independently associated with a 2.14-fold increased risk of death (95% CI: 0.48–9.65; *P* = 0.32) among infants born at 32∼37 weeks gestation, 5.73 (95% CI: 1.08–30.41; *P* = 0.04) at 28∼32 weeks gestation and 26.27 (95% CI: 6.22–110.98; *P* < 0.0001) at <28 weeks gestation. Accordingly, the result of May's study (15) showed, birth weight <1,500 g was independently associated with a 7.2-fold increased risk of death (95% CI: 4.8−10.9; *P* < 0.0001). Besides, Newborns of LBW were at higher risk of cognitive deficiency according to the study of Stevens et al. (30).

Although immature development and multiple pathological factors contribute to poor prognoses in preterm infants, preterm and LBW with BM are significantly correlated with death, no matter as independent prognostic predictors or confounding factors. And they are both important and potentially modifiable risk factors through optimal care in pregnancy and prevention of preterm delivery.

### 4.2 Seizures and coma

Seizures are generally considered as poor prognosis factors (19, 31). Ouchenir, et al. (27) reported, the BM infants (aged within 90 days) with seizures in hospital, comparing to those without seizures, were tend to have hearing loss (RR:8.4, 95% CI:1.0–72, *p* < 0.05), motor problem (spasticity, paresis) (RR:3.6, 95% CI:1.5–8.3, *p* < 0.05), developmental delay (RR: 4.2, 95% CI:1.4–13, *p* < 0.05) and death (RR:12, 95% CI:1.6–96, *p* < 0.05) at last encounter (the specific follow-up time is not given). But they only made univariate analyses. In Okike's study (26), 263 infants <90 days of age with BM were identified and seizures (OR, 7.06; 95% CI: 2.80–17.81) were independently associated with serious central nervous system complications (motor disorder or abnormal neurology, hydrocephalus, hearing loss or extradural collection requiring neurosurgical intervention). Similarly, this study did not specify the duration of follow-up, which cannot determine the relationship between convulsions and sequelae.

Since seizures have been reported as a presenting feature in 20∼50 percent of infants with BM, especially with gram-negative pathogens, and usually are focal, subtle or may be transient (32), more details in seizures should be taken into consideration in the prognostic analysis. In Klinger's study (19), 17 of 101 neonates with BM had moderate or severe disability at 1 year of age. Seizures were commonly found, presenting in majority of infants (92%) with adverse outcome and nearly half of the infants with good outcome. However, most of the infants (83.3%, 10/12) with duration of seizures for >72 h had poor prognosis. This can be explained by the fact that the persistence of seizures is due to underlying brain damage or raised intracranial pressure or electrolyte imbalance which all have been associated with poor outcome.

Besides, the presence of coma during the acute phase of BM was found associated with neonatal mortality in many studies (19, 21, 33), and was independently associated with 11.14-fold (95% CI: 3.01–40.87; *P* = 0.003) (17) to 31.85-fold (95% CI: 8.46–119.81, *P* < 0.0001) (26) increased risk of death.

Thus, as well-established proxies for severe illness, coma and persist seizures could be most important predictors for poor prognosis of BM.

### 4.3 Cerebrospinal fluid indexes

According to the included studies, high level of CSF protein was associated with poor prognosis independently, but with different cutoff values from 1.88 g/L (25) to 5.0 g/L (20), and even the same cutoff value (>5 g/L) with different risk folds (from OR: 171, 95% CI: 25.6–1,000 (20) to RR: 4.6, 95% CI:1.0–2.1 (27). In another two studies indicated higher CSF protein in the poor outcome comparing to good outcome group [3.45 (1.68, 5.82) vs. 1.51 (1.00, 3.14)] (24), [3.84(3.03) vs. 1.9 (1.7), *P* < 0.05] (21). Proteins gaining access to the CSF primarily reach the CSF by transport within pinocytotic vesicles traversing capillary endothelial cells. In BM, micro-organisms release endotoxins, teichoic acid, and other substances that trigger an inflammatory response with mediators such as white blood cells and tumor necrosis factor resulting in increasing protein levels in CSF (25). So, this can be explained that the elevated CSF protein was related to the intensity of the inflammatory response, as well as the production of high amounts of reactive oxygen species, which may cause impairment of lipids, proteins, carbohydrates or nucleic acids. Because of the high lipid content in the brain and low cerebral antioxidant defense, the central nervous system is particularly susceptible to the deleterious properties of oxidative stress (34, 35) hence poor outcome. Otherwise, CSF protein can also be elevated in noninfectious conditions, including conditions associated with obstruction of CSF flow, subarachnoid hemorrhage or a traumatic lumbar puncture (LP). In the study by Klinger (19), CSF protein concentrate was found irrelevant to adverse outcome at one year of age in infants with BM.

A study by Liu (36) showed CSF glucose < 1 mmol/L was an independent risk factor [OR: 11.38, 95% CI: 2.961–43.732] in predicting death and complications noted at discharge, but no long-term follow up information available. The CSF glucose level below 20 mg/dl and CSF/blood glucose ratio <0.2 have been shown to be associated with increased mortality (37). Other authors (19, 21) reported CSF/blood glucose ratio <0.5 was a predictive factor of mortality. But a low CSF glucose level was not associated with death in Kamoun's series (22). Besides, no one else has defined a best cutoff point yet.

Since CSF indexes vary according to age and influenced by various factors including collection time, detection time, specimen contamination, etc., the normal values are poorly defined (38, 39). The possibility of CSF protein and glucose as good predictors are challenged due to considerable overlap of values between infants with and without meningitis, and uncertainty value accuracy.

In addition, it was reported 44% (49/111) infants underwent repeat LP at a median of 5 (IQR: 3, 13) days after the LP that led to the diagnosis of BM, and WBC on the second spinal tap provided a cut-off value of 366 × 10^6^/L for predicting sequelae at discharge with sensitivity of 91% ad specificity of 88% (40). In a US study of 150 NICUs (41), 53% (118/221) infants with culture positive meningitis receiving ≥2 LPs during the treatment course, and the infants with repeat positive cultures on antibiotics were more likely to die (26% vs. 7%; *P* = .02), but did not report on other complications. But no significant difference in mortality was seen among the infants with a repeat negative culture compared with the BM infants with no repeat LP (*p* = 0.32). A survey of 109 pediatricians and neonatologists across northwest England found that 89 (82%) practitioners did not routinely repeat the LP in infants with BM unless clinically indicated (42).

As a result, the specific numerical values for CSF indexes that indicate a poor outcome remain uncertain. Further studies of the CSF manifestations of BM and their correlations with prognoses are needed.

### 4.4 Pathogenic factors

GBS (41.5%) and E. coli (24.1%) remained the predominant pathogens in BM infants in the first 90 days of life by summarizing all the included studies. Culture positive GBS cases in patients 0–3 months old between 1987 and 2016 were identified through Netherlands Reference Laboratory for BM with a Mortality of 8% (27/323) (43). In a Canadian study (27), the burden of GBS meningitis remains significant with a mortality rate of 14% with the 5 deaths occurring in term and preterm infants with a wide range of age at onset (1∼59 days of life) and the rate of cerebral infarcts was especially high among GBS meningitis patients (25%). The case fatality rate of 7% compared with that reported in other recent studies, namely 11% in the United Kingdom (4), 13% in France (7), and 15% in Taiwan (20). Moderate/severe disability was reported in 34% of infants with GBS meningitis, and 30% of the infants due to E. coli or other gram-negative bacilli (44).

While, in most studies of neonatal BM, the long-term outcomes were more common in survivors of Gram-negative bacterial (30). Harvey et al. (45) found sequelae in 58% of newborns with E. coli meningitis against 35% in those with GBS. May et al. (15) reported early onset meningitis caused by Gram-negative bacilli had a higher mortality than those due to other organisms (28.6% vs. 10.7%; OR 3.3, 95% CI 2.2 −4.9; *p* = 0.0001). These may be related to different demographic characteristics. It was reported E. coli meningitis was 7-fold more frequent in preterm than term infants (18) and almost 90% of the LBW infants with meningitis caused by Gram-negative organisms died (14). Neonatal Escherichia coli (E coli) meningitis results in significant morbidity and mortality (46). Tawny Saleh et al. (46) present a case of a premature infant with extensive central nervous system (CNS) injury from recurrent E coli infection. According to a recent Meta-analysis of bacterial pathogens (47), the frequency of GBS in neonates was highest in Europe and lowest in the Eastern Mediterranean region, with weighted means of 58.2% and 4.9%, respectively. E. coli and S. pneumoniae were the most common pathogens that caused BM in neonates in Africa (17.7% and 20.4%, respectively). Pneumococcal meningitis was reported independently associated with serious central nervous system complications (OR, 4.83; 95% CI: 1.33–17.58) and death (OR, 4.62; 95% CI: 1.19–17.91) (26), with the complications rate of 48% in pneumococcal meningitis, 21% in GBS, 19% in E. coli.

Despite the etiology of BM differs from region, era, age and birthweight etc. Gram-negative bacteria, especially E. coli, and GBS, S. pneumoniae, are all valuable for poor outcome.

Moreover, in a retrospective multicenter cohort study (48) on neonates with GBS meningitis followed-up 6 to 12 months period, abnormal cerebral ultrasound findings was related to adverse composite motor outcome (OR: 5.3, *p* = 0.017), extensive MRI lesions related to adverse composite cognitive outcome (OR: 7.0, *p* = 0.040), abnormal motor (OR 10.7, *p* = 0.040) and adverse composite motor (OR 12.6, *p* = 0.019).

In view of the diversity and complexity of predictors, some scholars have considered to build up prognostic models. In Klinger et al.'s prognostic tree models (19), seizures, coma, use of inotropes, and leukopenia were involved, which had a sensitivity of 68% and specificity of 100% at 12 h, and sensitivity of 88% and specificity of 99% at 96 h after admission. Haffner et al. (24) named Lasso model and concluded that clinical variables (seizures, pressor support) predicted death and neurodevelopmental impairment better than the neuroimaging or combined findings (area under the curve 0.88 vs. 0.79 and 0.83, respectively) and neuroimaging findings (cerebrovascular lesions, ventriculomegaly) predicted neurodevelopmental impairment better than clinical or combined findings (area under the curve 0.82 vs. 0.80 and 0.77, respectively) among survivors.

## 5 Conclusions

This systematic review *p* provides a preliminary exploration of prognostic factors for BM in young infants less than 90 days of age, and demonstrated several potential predictors. But there are notable differences in cutoff value, clinical factors selected and combined, and the final results related to poor outcome of BM in studies published so far. Further well-designed prognostic studies and quantitative analyses are needed to evaluate how the identified risk factors related with the prognosis of BM in young infants and how to be used to improve the clinical management of patients, counseling the parents about prognosis and planning ongoing and long-term care.

## 6 Limitations

In the process of article selection, some articles were excluded because of not written in English and the full-text could not be obtained, which would potential prognostic factors with significant value for predicting outcomes. It also was limited by the heterogeneity observed among the included studies in diagnostic criteria, definition of poor outcome, follow-up time and cutoff values.

## Data Availability

The original contributions presented in the study are included in the article/Supplementary Material, further inquiries can be directed to the corresponding author.

## Supplementary material

Supplementary Data Sheet Search strategy.

Supplementary S1. PRISMA 2009 checklist.

Supplementary S2. Diagnostic criteria, definitions of poor outcomes and main pathogens of BM in the included studies.
