# Supplementary material for: Clinical predictors of poor outcome of bacterial meningitis in infants less than 90 days: a systematic review
Source: Front Pediatr. 2024 Sep 19;12:1414778. doi: 10.3389/fped.2024.1414778 (PMC11448124; doi:10.3389/fped.2024.1414778)
Supplement: Supplementary Data Sheet [file Datasheet1.pdf]

Search strategy:（1950-2022）

PubMed: ((Meningitides, Bacterial[Title/Abstract] OR purulent meningitis[Title/Abstract] OR suppurative meningitis[Title/Abstract] OR Bacterial Meningitides[Title/Abstract] OR Bacterial Meningitis)[Title/Abstract] AND (Infants, Newborn[Title/Abstract] OR Newborn[Title/Abstract] OR Infant[Title/Abstract] OR Newborn Infants[Title/Abstract] OR Newborns[Title/Abstract] OR Newborn[Title/Abstract] OR Neonate[Title/Abstract] OR Neonates[Title/Abstract] OR Neonatal OR "<60 days" OR "<90 days")[Title/Abstract] AND (Prognoses[Title/Abstract] OR Prognostic Factors[Title/Abstract] OR Factor, Prognostic[Title/Abstract] OR Factors, Prognostic[Title/Abstract] OR Prognostic Factor[Title/Abstract] OR outcome[Title/Abstract] OR follow up[Title/Abstract] OR follow-up)[Title/Abstract]) **838** articles.

Embase: 'bacterial meningitis'/exp OR purulent AND meningitis OR suppurative AND meningitis AND ('newborn'/exp OR neonatal AND 'infant'/exp OR infants OR newborns OR 'neonate'/exp OR neonates) AND (prognoses OR prognostic AND factors OR prognostic AND factor OR prognosis OR 'outcome'/exp OR 'clinical outcome'/exp OR 'follow up'/exp) **563** articles.

Cochrane: Reviews matching "#3 - (prognoses OR prognostic OR prognosis OR 'outcome' OR 'follow up') AND (Bacterial Meningitides OR purulent meningitis OR suppurative meningitis OR Bacterial Meningitides) AND (Infants OR Newborn OR Infant OR Newborns OR Neonate OR Neonates)" **30** articles.
